# Laminin-332 alters connexin profile, dye coupling and intercellular Ca^2+ ^waves in ciliated tracheal epithelial cells

**DOI:** 10.1186/1465-9921-7-105

**Published:** 2006-08-02

**Authors:** Brant E Isakson, Colin E Olsen, Scott Boitano

**Affiliations:** 1 Department of Molecular Physiology and Biological Physics, University of Virginia School of Medicine, University of Virginia Charlottesville, Virginia 22908, USA; 2 Robert M. Berne Cardiovascular Research Center, University of Virginia School of Medicine, University of Virginia, Charlottesville, Virginia 22908, USA; 3 Arizona Respiratory Center, Arizona Health Sciences Center, University of Arizona, Tucson, Arizona 85724, USA; 4 Department of Physiology, Arizona Health Sciences Center, University of Arizona, Tucson, Arizona 85724, USA

## Abstract

**Background:**

Tracheal epithelial cells are anchored to a dynamic basement membrane that contains a variety of extracellular matrix proteins including collagens and laminins. During development, wound repair and disease of the airway epithelium, significant changes in extracellular matrix proteins may directly affect cell migration, differentiation and events mediated by intercellular communication. We hypothesized that alterations in cell matrix, specifically type I collagen and laminin α3β3γ2 (LM-332) proteins within the matrix, directly affect intercellular communication in ciliated rabbit tracheal epithelial cells (RTEC).

**Methods:**

Functional coupling of RTEC was monitored by microinjection of the negatively charged fluorescent dyes, Lucifer Yellow and Alexa 350, into ciliated RTEC grown on either a LM-332/collagen or collagen matrix. Coupling of physiologically significant molecules was evaluated by the mechanism and extent of propagated intercellular Ca^2+ ^waves. Expression of connexin (Cx) mRNA and proteins were assayed by reverse transcriptase – polymerase chain reaction and immunocytochemistry, respectively.

**Results:**

When compared to RTEC grown on collagen alone, RTEC grown on LM-332/collagen displayed a significant increase in dye transfer. Although mechanical stimulation of RTEC grown on either LM-332/collagen or collagen alone resulted in intercellular Ca^2+ ^waves, the mechanism of transfer was dependent on matrix: RTEC grown on LM-332/collagen propagated Ca^2+^waves via extracellular purinergic signaling whereas RTEC grown on collagen used gap junctions. Comparison of RTEC grown on collagen or LM-332/collagen matrices revealed a reorganization of Cx26, Cx43 and Cx46 proteins.

**Conclusion:**

Alterations in airway basement membrane proteins such as LM-332 can induce connexin reorganizations and result in altered cellular communication mechanisms that could contribute to airway tissue function.

## Background

The normal tracheal airway epithelial layer is composed primarily of pseudostratified ciliated, basal and secretory cells that maintain contact with each other and to a thin basement membrane [1]. Molecules comprising the airway extracellular matrix (ECM) consist of fibrous (e.g., collagens and elastin) and structural proteins (e.g., fibronectin and laminins) embedded in a hydrated polysaccharide gel containing several glycosaminoglycans (e.g., hyaluronic acid). Laminins are one of many basement membrane ECM molecules that can contribute to cell support and signalling of the airway epithelium [2]. Laminin was initially coined as a term to describe a single ECM protein but has come to encompass a family of heterotrimeric ECM proteins made up of single α, β and γ chains. To date, there are five α, three β and three γ chains that are known to form at least 16 laminin trimers and a variety of proteolytic fragments [3]. Laminins can be produced by lung epithelial cells, including bronchial cells [4,5]. A variety of laminins are expressed by lung epithelial cells during development and in adult tissue [6-11], including LM-332 (formerly Laminin-5) [5,12-14]. Differential LM-332/integrin interaction has been shown to be involved in airway epithelial wound responses in culture [15] and *in vivo *[13]. It is possible that the remodeling of ECM, including LM-332, by protein cleavage or structural changes can expose and/or eliminate ECM receptor binding sites and promote changes in signalling and cellular activity [16], however, direct studies on the effects of LM-332 on signalling of conducting airway cells are limited. In addition to ECM rearrangements, breach of the epithelial layer causes a redistribution of intercellular connections that are restored after reformation of the pseudostratified epithelial layer [17,18]. As a part of normal airway defense, epithelia coordinate cellular responses to prevent damage/toxicity. Airway epithelial cells rely on paracrine signalling and gap junctional communication to coordinate defence-related activities. Gap junctions are formed at points of cell-cell contact where each cell contributes a hexameric hemi-channel made up of connexins (Cx) [19,20]. Connexin proteins can convey unique permeability properties upon the gap junction channels, thus, alterations in connexin expression patterns can directly change the types of cell-cell communication between neighbouring cells, and contribute to local tissue response [21,22]. Direct studies on the effect of LM-332 on intercellular signalling of conducting airway epithelial cells have not been performed.

There is a complex pattern of connexin isoform expression in airway epithelial cells with at least eight different connexins expressed at various stages of differentiation and development: Cx26, Cx30.3, Cx31.1, Cx32, Cx37, Cx40, Cx43, and Cx46 [23-27]. Changes in connexin expression in upper airway epithelial cells have been associated with developing or post-injury airways *in vivo *[24,25]. *In vitro*, functional gap junctional intercellular communication has been traditionally monitored by transfer of low molecular weight fluorescent dyes, or by measurement of electrical conductance. Although these techniques are recognized as valuable experimental tools to identify cellular coupling, they do not always reflect transfer of physiologically significant molecules through gap junctions [21,26]. An alternative way to demonstrate gap junctional coupling in cultured airway epithelial cells is through monitoring of coordinated intracellular Ca^2+ ^concentration ([Ca^2+^]_i_) changes in response to mechanical stimulation of a single cell [28]. However, diffusion of second messenger molecules/ions through gap junctions is not the only way Ca^2+ ^waves can be propagated [29]. Following mechanical stimulation, cultured conducting airway epithelial cells can release nucleotides (e.g., ATP or UTP) into extracellular spaces resulting in the activation of Ca^2+ ^signalling pathways via plasma membrane purinergic receptors [30]. These pathways need not be mutually exclusive: we have shown in primary cultures of rat alveolar epithelial cells that addition of LM-332 to collagen matrices alters the mechanism of coordinating [Ca^2+^]_i _changes among neighbouring cells [26,31-34]. These changes in the coordination of Ca^2+ ^waves were accompanied by alterations of connexin isoform expression patterns and affected by cellular differentiation.

In this study we grew ciliated rabbit tracheal epithelial cells (RTEC) on substrates of LM-332/collagen or collagen alone and monitored functional dye coupling, propagation of intercellular Ca^2+ ^waves following mechanical stimulation, and alterations in connexin isoform expression. We found that, independent of the matrix substratum, ciliated RTEC were functionally coupled by low molecular weight dyes, although the incidence of dye coupling was increased by LM-332. Ciliated RTEC propagated intercellular Ca^2+ ^waves in response to mechanical stimulation on both matrices tested. However, cells grown on LM-332/collagen matrix propagated Ca^2+ ^waves via an extracellular nucleotide pathway whereas cells grown on collagen alone propagated Ca^2+ ^waves via gap junctions. Direct immunocytochemical staining of connexins showed a cellular rearrangement of at least three isoforms, Cx26, Cx43 and Cx46, in response to LM-332/collagen matrix. We suggest that similar changes of extracellular matrix proteins *in vivo *(e.g., during development, wound repair or disease) lead to changes in intercellular signalling that are important in coordinating upper airway epithelial tissue function.

## Methods

### Materials

Dulbeco's modified Eagle's media (DMEM), Hanks' Balanced Saline Solution, penicillin, streptomycin and amphotericin were from Gibco BRL (Grand Island, NY). Fura-2 and fura-2 acetomethoxy ester (fura-2AM) were from CalBiochem (La Jolla, CA). The connexin-mimetic peptides gap27 (amino acids SRPTEKTIFII; ADI, San Antonio, TX) and gap26 (amino acids VCYDKSFPISHVR; ADI) were used as gap junction inhibitors [26,35]. Apyrase, Lucifer Yellow (LY; MW = 457, Da; net charge = -2), flavin mononucleoside and ATP (cat #A 2383) were from Sigma Chemical (St. Louis, MO). LM-332 was from 804G cell culture supernatants [36]; the cell line was kindly provided by Dr. J.C.R. Jones, Northwestern University. Alexa 350 (MW = 350 Da; net charge -1) was from Molecular Probes (Eugene, OR). Goat anti-rat Cx26 and goat anti-rat Cx46 and primary antibodies were from Santa Cruz Biotechnologies (Santa Cruz, CA). The mouse monoclonal anti-Cx43 antibody was from Sigma Chemical. Alexa 488-labelled secondary antibodies were from Molecular Probes. All other chemicals were purchased from Fisher Scientific (Pittsburgh, PA) or Sigma Chemical and were of the highest analytical grade.

### Ciliated RTEC culture

Glass coverslips (15 mm) were coated with rat tail-collagen (primarily type I collagen), or rat tail-collagen supplemented with LM-332 rich 804G extract [36] (herein referred to as LM-332) as described [32]. RTEC cultures were prepared by methods described in [35]. Briefly, tracheas were removed from New Zealand White rabbits, the mucosa dissected and cut into small explants. After transfer to matrix-coated glass coverslips, the explants were placed in DMEM supplemented with NaHCO_3_, 10% fetal bovine serum and 1% antibiotic/antimycotic (penicillin, streptomycin, and amphotericin B), and cultured at 37°C in 5% CO_2_. Experiments were performed on 7 – 12 day old explant cultures. No morphological differences between cells grown on collagen matrix or LM-332/collagen matrix were observed (data not shown).

### Functional dye coupling

**Figure 1:**
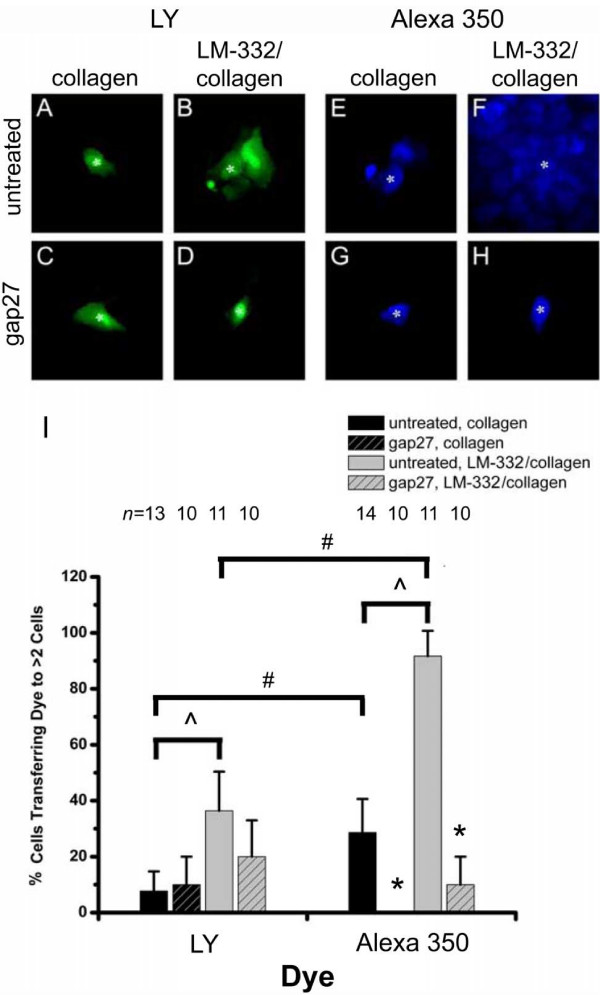
**Functional dye coupling in ciliated RTEC**. LY (A – D) or Alexa 350 (E – H) was microinjected into a single ciliated RTEC and allowed to diffuse for 5 min. Fluorescent micrographs represent typical experiments after microinjection into RTEC grown on collagen (A, C, E, G), or LM-332/collagen (B, D, F, H). Asterisks in fluorescent micrographs denote microinjected cells. The percent of microinjection experiments with dye transfer to greater than two cells after 5 min is graphed against the individual dye (I). "^" denotes a significant change in functional coupling between RTEC grown on different matrices; "*" denotes a significant change in functional coupling between RTEC grown on the same matrix with or without gap27; "#" denotes a significant difference in functional coupling as measured by different dyes; for all significance tests, P < 0.05. Values are ± standard deviation.

RTEC cultures were washed with Hanks' Balanced Saline Solution (HBSS: 1.3 mM CaCl_2_, 5.0 mM KC1, 0.3 mM KH_2_PO_4_, 0.5 mM MgCl_2_, 0.4 mM MgSO_4_, 137.9 mM NaCl, 0.3 mM Na_2_PO_4 _and 1% glucose additionally buffered with 25 mM HEPES, pH 7.4) and placed in 100-cm petri dishes containing HBSS at room temp. Eppendorf femptotips (Brinkmann, Westbury, NY) were backfilled with 10 mM Tracer dye (LY or Alexa 350) in 200 mM KCl. Dye was microinjected with an Eppendorf Micromanipulator 5171/Transjector 5426 into the cytoplasm of individual ciliated cells. Subsequent dye transfer was monitored on an Olympus IX70 inverted microscope (Melville, NY) with 20× objective in phase contrast during injections and in epifluorescence mode for dye coupling analysis. Cells were considered to be functionally coupled if two or more neighbouring cells displayed fluorescence within 5 min of dye injection. Dye coupling plots in Figure 1 display percent of experiments with functional coupling (i.e., dye present in more than 2 adjacent cells 5 min following microinjection). Images were captured 5 min post-injection with a CoolSnap camera (Roper Scientific, Tucson, AZ) onto a Apple Macintosh G4 computer (Cupertino, CA). Stock solutions of gap27 were made initially at 10 mg/ml in Phosphate Buffered Saline (PBS). Stock was diluted to a working concentration of 0.25 mg/ml (190 μM) in HBSS prior to experimentation. To obtain gap junction block, cells were incubated for a minimum of 45 min and up to 120 min. The nucleotidase, apyrase (50 U/ml in HBSS), was used to block paracrine signalling via ATP/UTP release [32]. Cells were washed with apyrase/HBSS for 1 – 30 min prior to experimentation.

### Measurement of intracellular Ca^2+ ^concentration ([Ca^2+^]_i_)

[Ca^2+^]_i _was calculated by ratiometric analysis of fura-2 fluorescence [37]. Fura-2 fluorescence was observed on an Olympus IX70 microscope after alternating excitation at 340 and 380 nm by a 75 W Xenon lamp linked to a Delta Ram V illuminator (Photon Technologies Incorporated (PTI), Monmouth Junction, New Jersey) and a gel optic line. Images of emitted fluorescence above 505 nm were recorded by an ICCD camera (PTI) and simultaneously displayed on a 23" colour monitor. The imaging system was under software control (ImageMaster, PTI) on an IBM clone computer. A change in [Ca^2+^]_i _was considered positive if the cell increased [Ca^2+^]_i _to 200 nM or more, a two to four fold change over resting values. Intercellular Ca^2+ ^waves were induced by mechanical stimulation of a single ciliated RTEC under piezo-electric control and performed with a glass micropipette (approx. 1 μm tip diameter) positioned near the apical membrane. The pipette was deflected downward for 150 msec to deform the cell membrane. If cell membranes were broken (as measured by loss of fura-2 dye) the experiment was not included in data analysis to prevent analysis of Ca^2+ ^wave propagation due to extracellular diffusion of intracellular contents [30,38]. Because the stimulated cell was included in analysis, a Ca^2+ ^wave of one cell represented no intercellular communication. In these experiments, the field of view varied, and was limited to between 20 and 40 cells (depending on individual culture). On occasion, wave propagation would encompass more than 20 cells (or exit the field of view). Ca^2+ ^wave propagation was given a total score of 20 cells in these cases. Because maximum numbers were imposed on cell counts, the number of cells participating in a Ca^2+ ^wave propagation in unblocked conditions are underrepresented. Each experimental paradigm was repeated on a minimum of 3 separate RTEC cultures (except gap26 inhibition studies).

### Reverse transcription polymerase chain reaction (RT-PCR) detection of connexin mRNA

To assay potential differences in mRNA expression of RTEC cells used in dye transfer and Ca^2+ ^imaging studies, tracheal explants were removed from 7–10 day old RTEC cultures and discarded. Total RNA from remaining outgrowth cells was isolated using the NucleoSpin RNAII kit (Clontech, Mountain View, CA) as per manufacturer's protocol. Isolated RNA (2 μg) was used as a template for reverse transcription with a First Strand cDNA Synthesis kit (Fermentas, Inc., Hanover, MD). Each 20 μl reaction mixture was prepared following the manufacturer's protocol with the exception of using both 0.5 μg of oligo dT primers and 0.2 μg of random hexamer primers in detection reactions. PCR reactions were carried out by mixing 2 μl of reverse transcription reaction, 5 μl of l0× PCR buffer containing 15 mM MgCl_2_, 1 μl of 10 mM deoxynucleoside phosphate mixture, 2 μM of PCR primer set, 0.25 μl of 5 U/μl Taq polymerase (Promega Corp., Madison, WI), and RNase/DNase free water up to 50 μl. An additional 7 μl of 25 mM MgCl_2 _(final concentration 5 mM) was added for Cx46 detection. Primer sequences for RT-PCR are shown in Table 1. Cx26 primer sequence was determined by inserting the NCBI rat connexin nucleotide sequences into the Primer 3 online program ; primer sequences for Cx43 [39], Cx46 [40], and actin [41] were adapted from published reports.

**Table 1:** Primer pairs for RT-PCR. Base sequences and product size for determining Cx26, Cx43, Cx46 and β-actin mRNA expression in RTEC

Gene	**Upstream Sequence**	**Downstream Sequence**	**BP**
Cx26	5'-CTGTCCTCTTCATCTTCCGC-3'	5'-TACGGACCTTCTGGGTTTTG-3'	306
Cx43	5'-CATTGGGGGGAAGGCGTGAGG-3'	5'-AGCGCACGTGAGAGATGGGGAAG-3'	400
Cx46	5'-GGAAAGGCCACAGGGTTTCCTGG-3'	5'-GGGTCCAGGAGGACCAACGG-3'	332
β-actin	5'-CGTGGGCCGCCCTAGGCACCA-3'	5'-TTGGCCTTAGGGTTCAGGGGGG-3'	242

### Immunocytochemistry of RTEC connexins

RTEC cultures were washed twice for 5 min with PBS and fixed with 4% paraformaldehyde for 10 min. Cell cultures were washed with PBS, incubated with PBS supplemented with 3% BSA, 5% serum (matched to secondary antibody source), 5% fish skin gelatin and 0.25% Triton X-100 (PBS-S) for 30 min, incubated overnight at 4°C with primary antibodies in PBS-S, and washed with PBS. Cell cultures were again incubated with PBS-S at room temperature, then incubated with secondary antibody in PBS-S for 1 hr, and washed thoroughly with PBS before being mounted for observation. Images were obtained on an Olympus Fluoview confocal microscope with a 60× WI objective (NA 0.9).

### Statistics

Functional dye coupling between individual cells were tested for equality and significant differences between variables using binary population proportion statistics. In comparisons between experimental paradigms, a statistical value of P < 0.05 was used to establish significance. Histograms display incidence of cell coupling with a particular dye within 5 min ± standard deviation. The mean number of cells participating in Ca^2+ ^waves under given conditions were compared between experiments by student t test. In comparisons between experimental paradigms a statistical value of P < 0.001 was used to establish significance. Histograms display number of cells participating in the Ca^2+ ^wave ± standard error.

## Results

### Dye coupling in RTEC cultures

To investigate if extracellular matrix proteins influence gap junctional communication in tracheal airway epithelial cells, we compared functional cell coupling after microinjection of tracer dyes into ciliated RTEC grown on matrices of collagen or LM-332/collagen. Representative fluorescent micrographs at 5 min following dye injections of individual ciliated RTEC are shown in Figure 1(A–H). Microinjection of LY into ciliated RTEC grown on collagen matrices resulted in successful coupling in only 7.7% of the experiments (Figure 1A,I) whereas ciliated RTEC grown on LM-332/collagen matrix displayed a significant increase in LY dye coupling (36.4% of the experiments; Figure 1B,I). In the presence of gap junction inhibitors (gap27, gap26 data not shown), LY coupling of ciliated RTEC grown on collagen matrix remained low (Figure 1C,I) while ciliated RTEC grown on collagen/LM-332 matrix displayed a reduced incidence of coupling (20%; Figure 1D,I). Similar to LY coupling in RTEC, Alexa 350 coupling was significantly higher in the RTEC grown on LM-332/collagen (91.7%; Figure 1F,I) than when grown on collagen (42.9%; Figure 1E,I). Also similar, functional coupling of Alexa 350 was significantly reduced in the presence of gap27 (or gap26; data not shown) on both matrices tested (Figure 1G–I). Despite these similarities, ciliated RTEC showed significantly increased coupling with Alexa 350 compared to LY whether grown on collagen or LM-332/collagen (Figure 1).

### Mechanically-induced Ca^2+ ^wave propagation in RTEC grown on LM-332/collagen matrices

**Figure 2:**
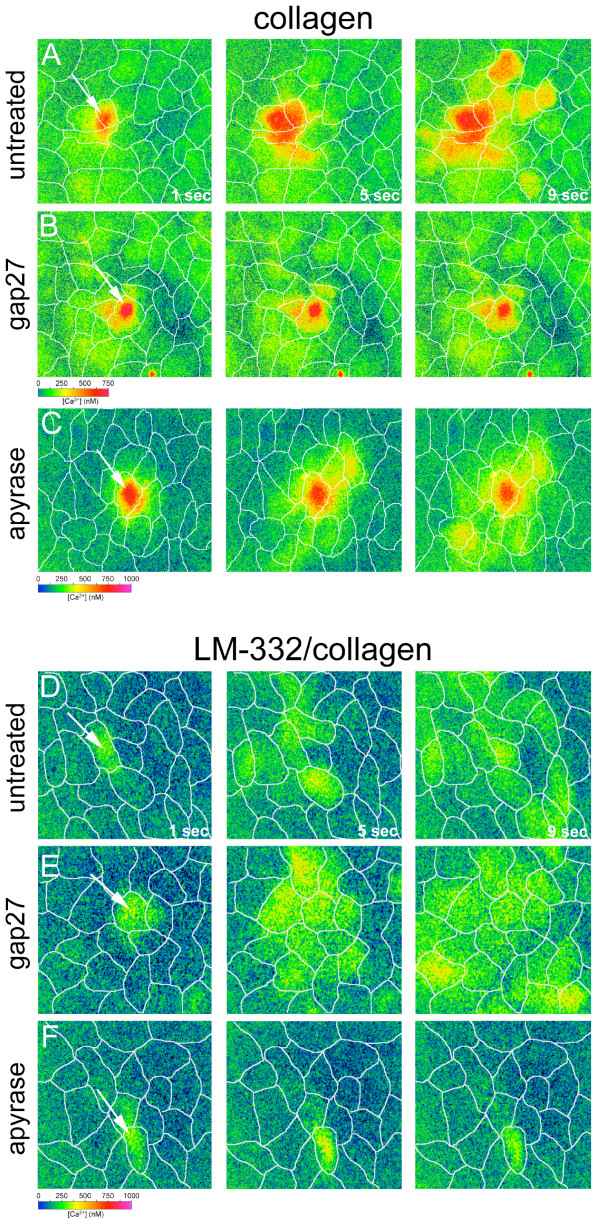
**Mechanically-induced Ca^2+ ^waves in RTEC plated on collagen or LM-332/collagen**. Pseudo-colour maps of increases in [Ca^2+^]_i _in RTEC over time after mechanical stimulation of a single ciliated RTEC (arrow) are shown. Each horizontal image sequence displays approximate [Ca^2+^]_i _concentrations (see inset) beginning at 1 sec and following at 5 and 9 sec after mechanical stimulation. White lines in each panel approximate cell boundaries. Two separate pseudo-colour scale bars are depicted for A, B; and C – F. The first three panels represent typical Ca^2+ ^waves in RTEC grown on collagen matrix under control conditions (A), treatment with gap27 (B), or treatment with apyrase (C). The last three panels represent typical Ca^2+ ^waves in RTEC grown on LM-332/collagen matrix under control conditions (D), treatment with gap27 (E), or treatment with apyrase (F). Although intercellular Ca^2+ ^communication is conserved in RTEC grown on collagen and LM-332/collagen matrices, the sensitivity to inhibitors show that the mechanism of communication is altered: RTEC grown on collagen propagate Ca^2+ ^waves via gap junctions, whereas RTEC grown on LM-332/collagen propagate Ca^2+ ^waves via extracellular nucleotide release.

**Figure 3:**
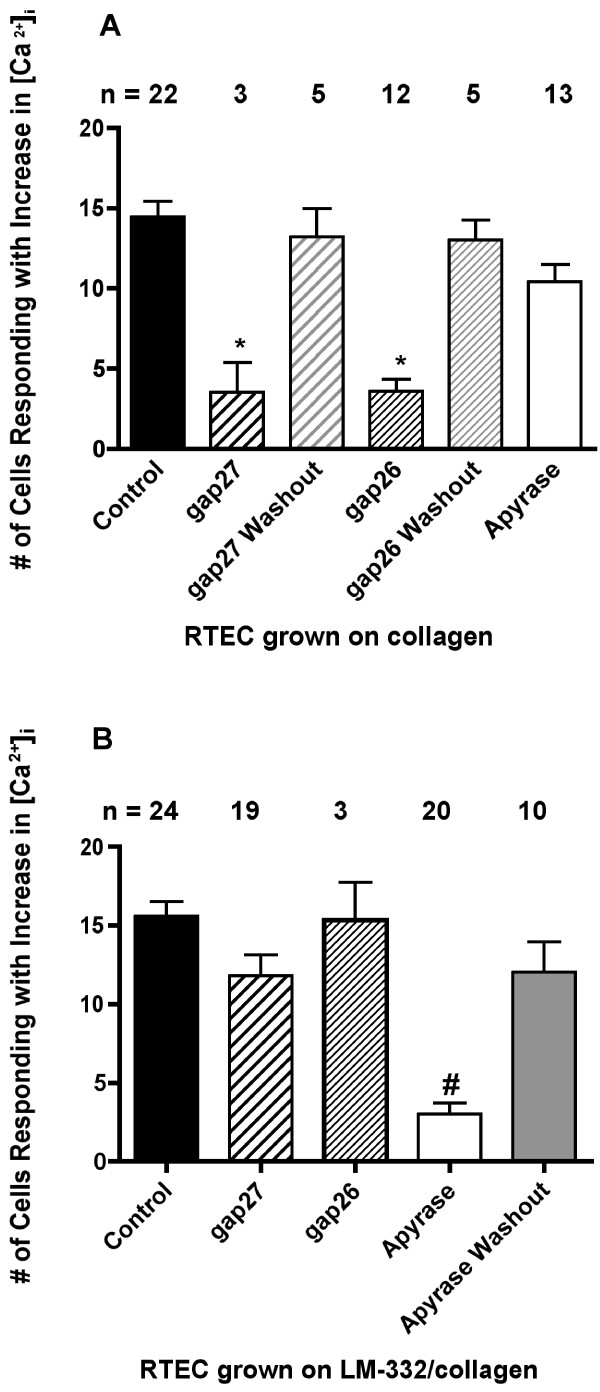
**Cell participation in mechanically-induced intercellular Ca^2+ ^waves in RTEC grown on collagen or LM-332/collagen matrices**. Cells responding with an increase in [Ca^2+^]_i _after mechanical stimulation are plotted against experimental paradigms described in Figure 2. A) Data are redrawn from [35] to illustrate gap junctional mediated Ca^2+ ^wave propagation in RTEC grown on collagen matrix. Under these conditions the gap junctional inhibitors gap26 and gap27 reversibly inhibit Ca^2+ ^wave propagation whereas the purinergic signalling inhibitor apyrase did not have a significant effect. B) When RTEC are grown on LM-332/collagen matrix, gap27 and gap26 had no effect on Ca^2+ ^wave propagation. In contrast, apyrase significantly inhibited propagation of Ca^2+ ^waves that were restored to control levels within 15 min of washout. RTEC cells grown on LM-332/collagen matrix propagated intercellular Ca^2+ ^waves via an extracellular purinergic pathway. Values are cells ± standard error. "*" indicates significant reduction from control (P < 0.01) washout (P < 0.01 for gap 26; P < 0.05 for gap27) and apyrase treatment (P < 0.05 for gap26). "#" indicates significant reduction in cell number as compared to any of the other treatments (P < 0.01 in comparison to gap26; P < 0.001 for all others).

In previous studies, mechanical stimulation of RTEC grown on collagen matrices has been shown to result in coordinated release of intracellular Ca^2+ ^in adjoining cells (intercellular Ca^2+ ^wave) via a gap junctional-dependent mechanism [28,29,35,42]. Representative mechanically-induced Ca^2+ ^waves of RTEC grown on collagen matrix under control conditions and in the presence of gap27 or a nucleotidase (apyrase) to block extracellular purinergic signalling are shown in Figure 2(A–C). On collagen matrices, mechanically induced Ca^2+ ^waves are restricted by gap junction inhibitors and not affected by nucleotidases ([35]; Figure 3A). To determine if the addition of LM-332 to a collagen matrix altered coordination of second messenger signalling between RTEC cells, we repeated these experiments with RTEC grown on LM-332/collagen matrices. Similar to RTEC grown on collagen, mechanical stimulation of a single ciliated RTEC resulted in an immediate increase in [Ca^2+^]_i _in the stimulated cell that was propagated to surrounding cells (Figure 2D). On average, 15.7 ± 0.9 cells participated in the mechanically-induced Ca^2+ ^wave (Figure 3B), a number not significantly different to that observed in cells grown on collagen matrix [35]. However, in contrast to results from RTEC grown on collagen, gap27 did not significantly lower the size of the mechanically-induced Ca^2+ ^wave (11.8 ± 1.3 cells; Figure 2E; Figure 3B). A second connexin mimetic peptide, gap26, also had no effect on RTEC Ca^2+ ^wave propagation (15.3 ± 2.4 cells; Figure 3B). An additional difference in the Ca^2+ ^wave propagation on LM-322/collagen matrices was the occasional initiation of [Ca^2+^]_i _changes in a participating cell at areas independent of cell-cell contact with an RTEC showing increased [Ca^2+^]_i _(data not shown), suggesting an extracellularly-mediated signalling event. RTEC cultures grown on LM-332/collagen matrix displayed increases in [Ca^2+^]_i _in response to external application of ATP or UTP (data not shown). In order to determine if purine release was a component of intercellular Ca^2+ ^wave propagation, mechanical stimulation was repeated in the presence of the nucleotidase, apyrase. The addition of 50 U/ml apyrase significantly reduced the number of cells participating in a mechanically-induced Ca^2+ ^wave in RTEC grown on LM-332/collagen matrices to 3.0 ± 0.7 cells (Figure 2F; Figure 3B). This reduction was reversed on washout of apyrase, where mechanically-induced Ca^2+ ^waves averaged 12.0 ± 2.0 cells (Figure 3B).

### Connexin isoform expression in RTEC grown on collagen and LM-332/collagen matrices

**Figure 4:**
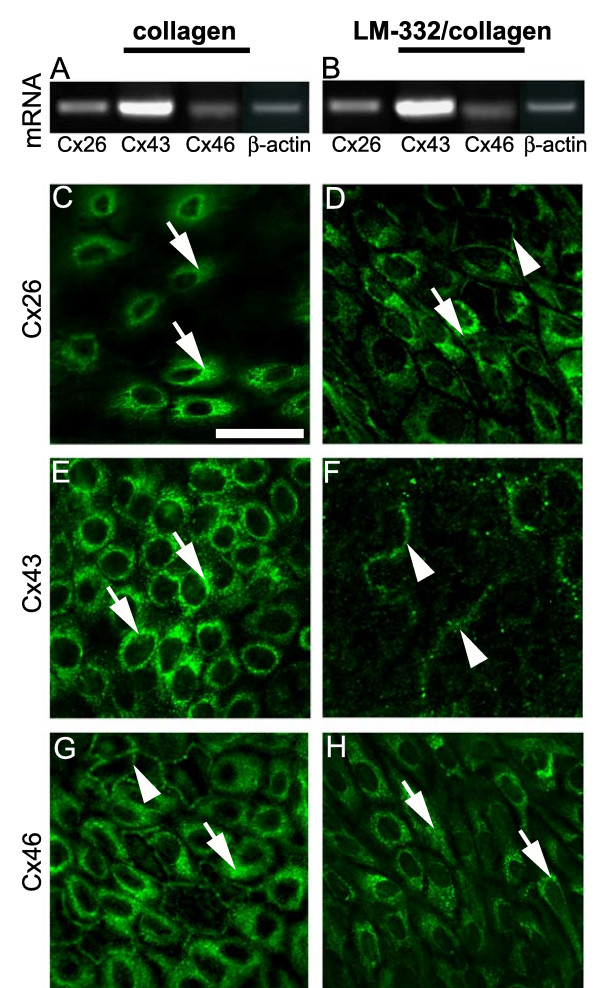
**Detection of connexin isoforms in RTEC by RT-PCR and immunocytochemistry**. RT-PCR (A, B) or immunocytochemistry (C-H) were used to identify connexin isoform expression changes between RTEC grown on collagen or LM-332/collagen. Total RNA was subjected to reverse transcription followed by PCR for Cx26, Cx43, Cx46 or β-actin (A, B). No differences in mRNA products from RTEC grown on either matrix were observed. Representative immunocytochemical micrographs of RTEC grown on collagen (C, E, G) or LM-332/collagen matrices (D, F, H) stained with antibodies against Cx26, Cx43, or Cx46 are shown. On the collagen matrices, all connexin isoforms display a perinuclear staining pattern, with a pericellular staining pattern also evident in the Cx46 micrograph. On the LM-332/collagen matrices, a noticeable shift in pericellular staining is evident in Cx26 and Cx43 micrographs, whereas the most evident staining of Cx46 is perinuclear. Growth of RTEC in the presence of LM-332 alters the spatial pattern of connexin isoform expression. Arrowheads denote pericellular staining and arrows denote perinuclear staining. Bar in C represents 20 *μ*m and is relevant to C – H.

Because we detected differences in functional and physiological coupling in RTEC grown on differing matrices, we used RT-PCR to detect possible changes in connexin mRNA expression of three known lung epithelial connexins: Cx26, Cx43 and Cx46. No discernable matrix associated differences in connexin mRNA expression were observed (Figure 4A–B). We next used immunocytochemistry to evaluate if spatial distribution of connexin isoforms were altered by extracellular matrix (Figure 4C–H). RTEC grown on collagen matrices displayed a perinuclear staining pattern for all three connexin isoforms tested (Figure 4C,E,G) with intermittent pericellular staining in the Cx46 micrographs (Figure 4G). RTEC grown on a LM-332/collagen matrix displayed distinctly different spatial patterns of staining for each connexin tested (Figure 4D,F,H). Although Cx26 micrographs displayed perinuclear staining, an additional pericellular pattern emerged (Figure 4D), whereas the Cx43 staining pattern was almost entirely pericellular (Figure 4F). In contrast to Cx26 and Cx43, the pattern for Cx46 lost the distinct pericellular stain and displayed mostly a perinuclear pattern (Figure 4H).

## Discussion

The airway epithelium relies on intercellular communication to coordinate cellular behaviour into tissue function. Such communication is sensitive to changes in the local environment. In this study we used fluorescent dye transfer and intercellular Ca^2+ ^wave coupling assays to elucidate alterations in cell-cell signalling of ciliated RTEC grown on either a collagen or a LM-332/collagen matrix. Diffusion of negatively charged low molecular weight dyes between cells was significantly increased in the RTEC grown on LM-332/collagen matrices. In contrast to the significant increases in dye coupling, gap junctional coupling for physiologically-relevant second messenger molecules that help to coordinate intercellular Ca^2+ ^waves was severely restricted when cells were grown on the LM-332/collagen matrix. Direct analysis of three connexin isoforms – Cx26, Cx43 and Cx46 – displayed a spatial redistribution coincident with matrix and functional/physiological coupling changes. Taken together, ciliated epithelial cells have distinct intercellular signalling pathways that are responsive to alterations of ECM proteins such as those occurring during development, or in response to wounding or disease.

Molecules comprising the airway ECM consist of both fibrous (e.g., collagens and elastin) and structural (e.g., fibronectin and laminins) proteins. Laminins are one of many basement membrane extracellular matrix molecules that can contribute to cell support and signalling of the developing airway [2,5,7,9,12]. The laminin isoform LM-332 can be remodelled in the conducting airway during injury or disease [6,43,44]. We have shown that LM-332 has profound effects on cell signalling, development and morphology in primary cultured alveolar epithelial cells [26,31-34]. In the bronchial airway epithelium, LM-332 can contribute to hemidesmosome formation [5], however, specific effects of LM-332 on cellular physiology of conducting airway epithelial cells remain ill-defined.

Direct cellular coupling through gap junctions has been traditionally monitored by transfer of low molecular weight fluorescent dyes or by measurement of electrical conductance. Both ciliated and aciliated RTEC have been shown to be electrically coupled [45]. Initial experiments reported herein focussed on the effects of LM-332 on cell-cell coupling between RTEC using fluorescent tracer molecules. In our findings, RTEC grown on collagen were poorly coupled with LY and showed a low but significantly higher coupling with Alexa 350. When RTEC were grown on collagen matrices that included LM-332, significant increases in both LY and Alexa 350 dye transfer were observed. These shifts in dye coupling in response to LM-332 matrices are similar to increased gap junctional permeability of calcein (MW 622 Da; net charge = -3) in keratinocytes grown on LM-332 and collagen matrices [46]. The fact that increase in gap junctional permeability to fluorescent markers after growth on LM-322 occurs across cell types may represent a general response to altered matrices.

Although dye coupling techniques are recognized as valuable experimental tools to identity functional gap junctions, it has become increasingly clear that gap junctions made of different connexin isoforms can also allow the differential transfer of physiologically relevant molecules [21,47,48]. To evaluate potential differences in the transfer of physiologically significant molecules, we initiated mechanically-induced Ca^2+ ^waves between RTEC grown on collagen or LM-332/collagen matrices and used specific inhibitors to identify intercellular signalling pathways. A role for gap junctions in mechanically induced Ca^2+ ^waves in RTEC grown on collagen matrices has been firmly established [28,29,35,42,49-51]. In this model, mechanical stimulation induces both the opening of Ca^2+ ^channels in the plasma membrane and an increase in 1,4,5-inositol trisphosphate (IP_3_) concentrations in the stimulated cell [50,51] that can further increase [Ca^2+^]_i _of the stimulated cell through release of Ca^2+ ^from intracellular stores. The changes in [Ca^2+^]_i _in adjacent cells is through a gap junctional mediated, IP_3_-dependent Ca^2+ ^release [29,35,42,51]. A role for paracrine signalling via mechanically-induced ATP or UTP release in primary cultured mouse and human airway cells has been established also [30,52,53]. In this model, mechanical stimulation induces release of nucleotide triphosphate that diffuses in the extracellular environment and binds to purinergic receptors on adjacent cells, activating cellular signals that lead to increases in [Ca^2+^]_i_.

In this study we show that when RTEC are grown on a LM-332/collagen matrix, mechanically-stimulated Ca^2+ ^waves are conserved. However, inhibitor studies are consistent with a shift in the mechanism of coordination of Ca^2+ ^changes to a paracrine/purinergic signalling pathway. Although cultured RTEC cells grown on collagen [38,54,55] or LM-332/collagen (data not shown) can respond to extracellular ATP or UTP by increasing [Ca^2+^]_i_, it is only the RTEC grown on LM-332/collagen that utilize purinergic signalling in response to mechanical stimulation to coordinate [Ca^2+^]_i _changes. This pronounced switch in communication mechanisms in RTEC cultures in response to LM-332 suggests that differences in mechanically-induced Ca^2+ ^communication between rabbit [28,29,35,42,49-51] and mouse or human airway epithelial cell cultures [30,52,53] may not be due to species-specific differences in airway signalling. Given the extensive remodelling of matrix during development, wound response and disease, mechanisms of cellular communication might also be "remodelled" at these crucial times for coordinated airway epithelial tissue function.

In an attempt to determine specific changes in gap junctions that contributed to the observed alterations in dye and second messenger coupling in RTEC, we examined directly the expression and spatial organization of three connexin isoforms: Cx26, Cx43 and Cx46. All of these isoforms showed mRNA and protein expression in RTEC after growth on either matrix, however, spatial distribution of each of these connexin isoforms was dependent on matrix. On LM-332/collagen matrices Cx26 and Cx43 isoforms were more prominent and Cx46 was less prominent at the cell membrane. These results are not entirely consistent with our previous report that examined connexin isoforms in RTEC grown on collagen [42]. Using rabbit polyclonal antibodies we detected only a slight pericellular Cx26 staining pattern, an extensive pericellular staining of Cx32, and a lack of Cx43 isoform staining. Our experience with multiple antibodies for connexin isoforms [56] allowed for a more direct probe of connexins in RTEC reported herein. The establishment of Cx26 or Cx43 gap junctions at the plasma membrane in RTEC grown on LM-332/collagen matrices may account for increased dye coupling; both Cx26 and Cx43 have been shown to increase LY transfer in transfected HeLa cells [57]. In contrast, in experiments directed at testing isoform second messenger transfer through gap junctions, neither Cx26 nor Cx43 was as efficient as Cx32 in allowing transfer of IP_3 _after microinjection [48]. Similar to what is shown here, increases in dye transfer do not necessarily correspond to second messenger transfer via gap junctions. Because gap junctions made of Cx32 allow for transfer of IP_3 _and Cx32-specific antibodies can directly inhibit Ca^2+ ^wave propagation in RTEC grown on collagen [42], we suspect changes of this connexin isoform also occur after RTEC are grown on LM-332/collagen matrices. Additionally, we cannot rule out that Cx46 rearrangements shown herein contribute to the observed changes in second messenger coupling. As noted for dye coupling experiments above, there is precedence also for the regulation of connexin expression in response to LM-332 in the extracellular matrix [31,32,46]. In primary cultured alveolar epithelial cells, LM-332/collagen induced a similar change in mechanism of Ca^2+ ^communication to that observed in RTEC cultures presented in this study [31,32]. In addition, an upregulation of Cx26 and a downregulation of Cx43 were reported in these cells, as well as significant changes in cell morphology [31,32,34]. In RTEC, no apparent changes in cellular morphology, connexin protein or mRNA expression were noted when LM-332 was included in the collagen matrix. The observed LM-332 induced changes were more similar to those seen in transfected CHO cells, where a distinct translocation of Cx43 from the cytoplasm to the plasma membrane occurred in the presence of LM-332 [46].

In summary, the observed connexin re-organization in RTEC grown on collagen or LM-332/collagen matrices does not inhibit fully cellular coupling as demonstrated by the LY and Alexa 350 dye transfer, but shifts the ways in which the cells communicate an increase in [Ca^2+^]_i_. It should not be discounted that a subtle change in the pattern of Ca^2+ ^signalling can have a significant change on the cellular physiology of the signal [58]. In addition to the effects of changes in Ca^2+ ^signalling on cellular physiology, the direct transfer of several other second messenger/small metabolites could also be altered after connexin re-organization. ATP, ADP, glutathione, glutamate, IP_3_, cAMP, cGMP have all been shown to have altered permeability through gap junctions made up of different connexin isoforms [21,47,48,59]. Thus, the documented changes in LM-332 during wounding, development or pathology could directly affect intercellular communication within the conducting airway epithelium to coordinate a variety of tissue responses.

## Conclusion

LM-332 alters cellular signalling and gap junction permeability in rabbit tracheal epithelial cells that are associated with a change in connexin isoform organization. Unlike previous reports in rat alveolar epithelial cells, changes in permeability and connexin isoform expression occurred without obvious changes in cell morphology. We conclude that LM-332 may be an ECM signal to help shape intercellular communication and tissue function in the conducting airway.

## Competing interests

The author(s) declare that they have no competing interests.

## Authors' contributions

BEI contributed cell culture, initial microinjection experiments and was responsible for all immunocytochemistry experiments. CEO contributed to cell culture, microinjection experiments and was responsible for RT-PCR. SB contributed to cell culture and was responsible for digital imaging of [Ca^2+^]_i_. All authors contributed to design of experiments, drafting of the manuscript and approved of the final manuscript.
